# Manual physical balance assistance of therapists during gait training of stroke survivors: characteristics and predicting the timing

**DOI:** 10.1186/s12984-017-0337-8

**Published:** 2017-12-02

**Authors:** Juliet A. M. Haarman, Erik Maartens, Herman van der Kooij, Jaap H. Buurke, Jasper Reenalda, Johan S. Rietman

**Affiliations:** 1 Roessingh Research and Development, Roessinghsbleekweg 33b, 7522 AH Enschede, the Netherlands; 2 Department of Biomechanical Engineering, University of Twente, Drienerlolaan 5, 7522 NB Enschede, the Netherlands; 3 Roessingh Research and Development, Roessinghsbleekweg 33b, PO Box 310, 7500 AH Enschede, the Netherlands

**Keywords:** Stroke rehabilitation, Gait training, Technical requirements, Balance-assisting characteristics, Algorithm development, Behavior prediction, Correction forces

## Abstract

**Background:**

During gait training, physical therapists continuously supervise stroke survivors and provide physical support to their pelvis when they judge that the patient is unable to keep his balance. This paper is the first in providing quantitative data about the corrective forces that therapists use during gait training. It is assumed that changes in the acceleration of a patient’s COM are a good predictor for therapeutic balance assistance during the training sessions Therefore, this paper provides a method that predicts the timing of therapeutic balance assistance, based on acceleration data of the sacrum.

**Methods:**

Eight sub-acute stroke survivors and seven therapists were included in this study. Patients were asked to perform straight line walking as well as slalom walking in a conventional training setting. Acceleration of the sacrum was captured by an Inertial Magnetic Measurement Unit. Balance-assisting corrective forces applied by the therapist were collected from two force sensors positioned on both sides of the patient’s hips. Measures to characterize the therapeutic balance assistance were the amount of force, duration, impulse and the anatomical plane in which the assistance took place. Based on the acceleration data of the sacrum, an algorithm was developed to predict therapeutic balance assistance. To validate the developed algorithm, the predicted events of balance assistance by the algorithm were compared with the actual provided therapeutic assistance.

**Results:**

The algorithm was able to predict the actual therapeutic assistance with a Positive Predictive Value of 87% and a True Positive Rate of 81%. Assistance mainly took place over the medio-lateral axis and corrective forces of about 2% of the patient’s body weight (15.9 N (11), median (IQR)) were provided by therapists in this plane. Median duration of balance assistance was 1.1 s (0.6) (median (IQR)) and median impulse was 9.4Ns (8.2) (median (IQR)). Although therapists were specifically instructed to aim for the force sensors on the iliac crest, a different contact location was reported in 22% of the corrections.

**Conclusions:**

This paper presents insights into the behavior of therapists regarding their manual physical assistance during gait training. A quantitative dataset was presented, representing therapeutic balance-assisting force characteristics. Furthermore, an algorithm was developed that predicts events at which therapeutic balance assistance was provided. Prediction scores remain high when different therapists and patients were analyzed with the same algorithm settings. Both the quantitative dataset and the developed algorithm can serve as technical input in the development of (robot-controlled) balance supportive devices.

## Background

Stroke survivors with a Functional Ambulation Category (FAC) of 3 often experience reduced balance control and difficulties with independent ambulation [1]. Physical therapists focus on improving these aspects in rehabilitation therapy, for instance by training tasks that specifically relate to Activities of Daily Living (ADL’s) such as overground walking in and around the house [2, 3]. During these training sessions, therapists continuously need to supervise patients when they walk. When patients lose their balance, therapists provide manual physical balance assistance to the body in the form of small corrective forces. In any given situation, therapists consider patient-specific examination findings to determine if and when balance assistance is needed, such as a patient’s specific muscle strength, isolated movement capacity, reaction or movement time deficits, co-morbid sensory loss, coordination deficits, as well as, fatigue status and fall history. Providing balance assistance not only allows patients to continue their training safely, it also lets them experience the boundaries of their abilities without actually falling. Such a process of experiencing trial-and-error in (re)learning motor tasks is commonly referred to as error-based training, a concept often applied in stroke rehabilitation [4]. The applicability of this concept was confirmed in observations during training sessions by the authors and by personal communication with therapists, who state that an optimal tradeoff between safety of the patient and physical manual balance assistance by the therapist is critical. When therapists provide assistance too soon, patients might not learn from their mistakes as imbalance is already corrected by the therapist before it is noticed by the patient. On the contrary, when therapists provide assistance too late, more corrective force is needed to stabilize the patient, thereby creating a possibly dangerous situation in which a therapist is not able to prevent the patient from falling. It has been shown that such (sensory) feedback is important in the learning process of patients [5, 6], especially when the patient is able to link this information to his/her body movements [7].

Even though gait training was found to be effective for stroke survivors in terms of regaining functional independence [8, 9], the one-on-one contact with the patient and the constant need for supervision makes this type of therapy time consuming, labor intensive and expensive in terms of healthcare costs. The burden on health care is expected to increase even further in the near future, limiting rehabilitation time and potentially its effectiveness for patients. Patients benefit from, among other aspects, a training environment where sufficient training hours at a suitable training intensity can be made [10]. A solution that might positively contribute to this aspect is the use of (robotic) devices that support balance [11, 12], with which patients can undertake additional training hours in a self-administered training environment. The intuitive and effective training method of therapists, and their complex integration of knowledge of the patient’s abilities might be of great importance in the acceptance and effectiveness of such a device. Therefore, a first step in designing such a particular training device is to investigate this complex behavior and the possibilities to convert this information into a robotic device. Previous work by Galvez et al. was performed with a similar goal in mind [13]. The study intended to quantify and analyze the interaction forces between the physical therapist and the patient’s hips and legs, while performing gait training that required continuous support by more than one therapist. However, no quantitative data is currently available that describes how a single therapist provides intermittent manual balance assistance to patients that are able to walk for short distances, nor is data available that describes when therapists intuitively decide that patients need support.

Therapeutic balance-assisting forces could be provided at many locations on the body. Since no data is available on this, observations during training sessions by the authors and by personal communication with therapists have identified that the iliac crest is a preferred point of contact. Possibly as this allows accurate control of the center of mass (COM), an important parameter when it comes to balance. Additionally, a large number of existing fall detection systems [14–18] reflect that a relation exists between acceleration signals measured at the COM and the likelihood for falls. These fall detection systems measure accelerations of the COM and process this data by the use of specific algorithms in order to determine if and when the subject fell. The algorithms were validated by comparison of the detected falls from the recorded data with the actual falls of the subjects during the measurements. The studies all showed the ability to distinguish true falling events from other activities based on acceleration data, and a sensitivity >80% and specificity of 100% was shown by Mathie et al. [15]. COM accelerations therefore seem to be highly related to falls. Other fall detection systems exist that use acceleration data of different body parts (i.e. trunk, thigh, head), but Kangas et al. [19] found that measurements from the waist and head were more useful for fall detection compared to the other body parts.

Predicting the intuitive and complex behavior of therapists in providing balance assistance logically depends on many more aspects than is the case in the predication of falls. Yet, given the relation between COM acceleration and falls as described above and the pelvis as the preferred place of therapists to provide balance assistance, we assume that a change in the acceleration of the patient’s COM plays an important role in the decision of therapists to provide balance assistance. Although it might not capture all the moments in time at which balance assistance takes place, it is a first step in the development of the previously mentioned robotic training device. This paper therefore aims to develop an algorithm that predicts the timing of therapeutic balance assistance during overground walking, based on the accelerations of the patient’s sacrum. Note that, in contrast to existing fall detection algorithms, the developed algorithm aims at detecting events of therapeutic balance assistance rather than to detect actual falls of the subjects. By comparing the predicted events of physical balance assistance with the actual therapeutic events, a measure is provided that validates the predictive abilities of the algorithm. Moreover, in order to provide insight into how therapists support patients during gait training, this paper will be the first to provide a step towards quantifying therapeutic balance-assisting forces. It will quantify balance assistance in terms of the anatomical planes in which force is provided, the duration of the force and the amount of force necessary to regain or retain the balance of the patient during overground walking. Aside from the general insight that this information gives into the behavior of therapist during gait training sessions, both the algorithm as well as the quantitative data can serve as a first step in setting up technical requirements for robotic balance gait training devices.

## Method

### Subjects

Eight male stroke survivors (age = 58 ± 6 years, height = 1.82 ± 0.06 m, weight = 84 ± 7.2 kg) were recruited for the overground walking task. Subjects were both recruited from Roessingh Rehabilitation Hospital in Enschede, The Netherlands, and physiotherapy practice PMI Rembrandt in Veenendaal, The Netherlands. Subjects were included if they met the following inclusion criteria: (1) Stroke survivor (either (sub-)acute or chronic); (2) Able to walk for a short distance (10 m) without a walking aid, but with physical supervision of a therapist; (3) Able to understand and execute instructions of the walking tasks.

This study was approved by the local Medical Ethical Institutional Review Board and methods conformed to the Declaration of Helsinki. All subjects gave written informed consent prior to participation.

### Equipment

**Fig. 1:**
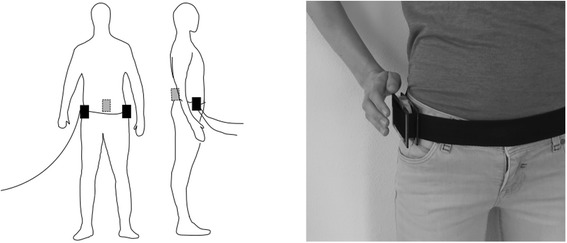
(Schematic) representation of the measurement systems on the body. Left: Measurement systems positioned at the body: Force/Torque sensors were (with a hip belt) positioned at both sides of the hips (black blocks). One IMU sensor (grey block) was positioned with adhesive skin tape to the sacrum of the subject. Right: Positioning of the Force/Torque sensor to the body

Subjects were instrumented with two Force/Torque sensors and one inertial measurement unit (see Fig. 1).

To capture the balance-assisting force characteristics of therapists, the two wired 6 Degree of Freedom Force/Torque sensors (Mini45 F/T sensor, ATI Automation Industrial, Apex, NC, USA) were fixated on the left and right side of a belt that was worn around the pelvis of the patient (Fig. 1). Specifically, the sensors were positioned at the location of the iliac crest. Prior to the start of measurement trials, interviews were conducted with the patient’s physical therapist and entire physical therapy treatment sessions were observed during which gait training sessions occurred. These sessions have revealed that the iliac crest is the preferred location to provide balance assistance to the patient. The belt with the sensors were oriented such that walking direction of the patient equaled the x axis of the sensor for each subject. The force sensors were connected to a power supply that was connected to a Data Acquisition (DAQ) card (National Instruments, Austin, TX, USA) in order to acquire the signals in Simulink (MathWorks, Natick, MA, USA).

A wireless inertial magnetic measurement unit (IMU) (MTw sensor, Xsens Technologies B.V., Enschede, the Netherlands), consisting of an accelerometer, gyroscope and magnetometer, was positioned at the sacrum to capture sacral motion throughout the measurement. The accelerometer signal (60 Hz) was specifically measured at the sacrum as this location closely resembles COM kinematics [20]. The IMU was mounted with adhesive skin tape to the lower back of the subject during overground walking training. Data recording was performed through MT Manager software (Xsens Technolgies B.V., Enschede, the Netherlands).

To synchronize both measurement systems, a switch was manually pressed on one of the force sensors (leading to a peak in this data-signal), at the same time triggering the IMU software to start recording. Force data was cut afterwards in MATLAB (MathWorks, Natick, MA, USA), based on this synchronization peak.

### Protocol

Measurements took place during a regular gait training session, and lasted no longer than 30 min for each patient. Patients were asked to arrive 15 min prior to the start of the measurement in order to mount the sensors correctly onto the body. Subjects walked at a self-selected walking speed and a physical therapist walked behind the patient to supervise and physically support the patient when necessary. Therapists were instructed to correct the balance of the patients at the location of the force sensors (at the iliac crest). Therapists did not have their hands on the patients at times other than when corrective forces were being applied.

**Fig. 2:**
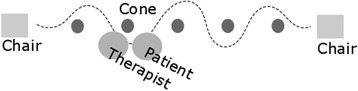
Schematic representation of the measurement set-up. Legend: Chairs were positioned 10 m from each other. In between the chairs were five cones, such that patients had to walk around them. Therapist walked behind the subject and only provided assistance when the patients was unstable

Similar to conventional gait training sessions, subjects were asked to slalom (zigzag movement) around cones and walk in a straight line during the measurements, in order to represent training of ADL tasks. Patients that only needed few therapeutic balance-assisting force corrections were given an additional cognitive task (e.g. count down in increments of three) in order to provoke more postural instabilities during the measurements. Tasks were repeated in a random order until the end of the training time was reached, or until the patient was tired. Training took place on a level ground walkway of approximately 10 m with a chair on both sides so that patients could rest in between the measurements (see Fig. 2). As the force sensors were connected with wires, a trolley was used to guide the wires and transport the laptop alongside the patient.

When therapists provided corrective forces at a location other than the force sensors (e.g. at the shoulder or the trunk of the patient), no force characteristics could be captured by the force sensors. However, the moment in time of these balance-assisting events was still captured, as a timestamp was manually created in Simulink by one of the researchers that logged the time and the location of these events.

In order to obtain patient characteristics, Berg Balance Scale (BBS), Functional Ambulation Category (FAC), ten meter walking test (10MWT), Motricity Index (MI) and Dynamic Gait Index (DGI) were performed within one week after the measurement by the treating physical therapist of the patient.

### Data analysis

#### Balance-assisting force characteristics based on the force sensor data

Force data was filtered over all three axes with a 2nd order Butterworth, low-pass filter with a cutoff frequency of 5 Hz. Baseline offset was removed during a static condition prior to the start of each measurement and the resultant force between the sensor that was mounted on the left and right side of the body was calculated over all axes separately. For all measurement trials, the start and stop time of each individual therapeutic balance-assisting event was manually selected. All measurements were checked and synchronized in correspondence with video recordings that were taken during the measurements. Subsequently, the maximal force and the duration of the therapeutic corrective forces were calculated for each event individually. Maximal force was presented as the absolute value, irrespective of the sign (positive or negative) of the correction applied.

#### Predicting the timing of therapeutic balance assistance based on acceleration data of the sacrum

Accelerometer data of the sacral IMU was processed in MATLAB, for each subject and measurement trial individually. The resultant acceleration signal of the X, Y and Z axes of the sensor was used for analysis, making the data independent of sensor orientation on the sacrum. The mean resultant value was subtracted from the data, such that gravitational acceleration was removed and free acceleration data remained. The periods in time where a patient was not moving before the start and after the stop of each measurement were cut off, leaving only the actual movement data to be analyzed.

**Fig. 3:**
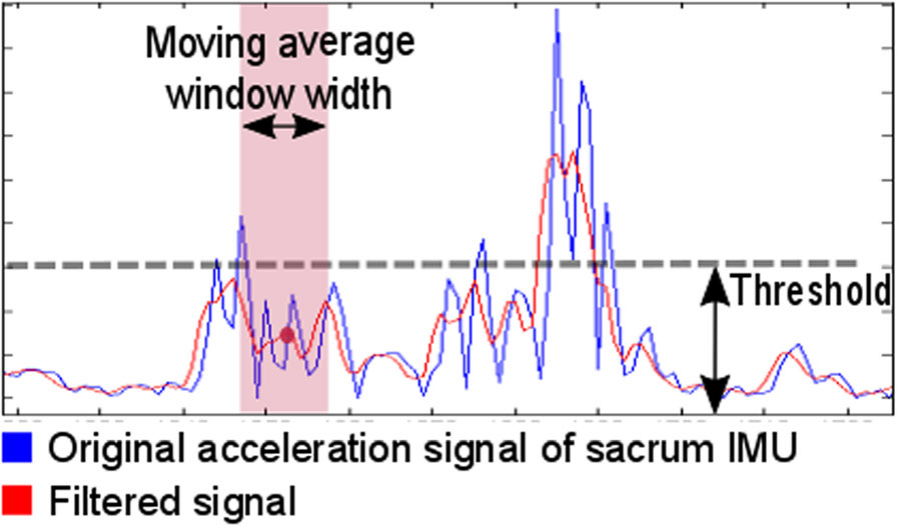
A moving average filter moves along the original signal (blue line), calculating an average value (red dot) of the data points within the window (red square). The red line represents the result of this. Each time the red line was above the pre-set threshold value, the moment in time was marked as an outlier

The algorithm that was built is based on the assumption that deviations in the sacral signal (for instance: relatively long periods in time where high accelerations in the signal are visible) represent the moments in time were therapeutic balance assistance takes place. Outlier detection was used to identify these time points. Interquartile ranges (IQR) and individual quartiles (e.g. Q1 - Q4) were calculated on the remaining dataset and were used to define the threshold for outlier detection of each subject and measurement trial individually [21]. Additionally, a moving average window (‘δ’, seconds) was applied to calculate the average value of the data within a specified window width. A visual representation of the method used is presented in Fig. 3. A threshold was set at Q_3_ + α*IQR according to Tukey’s method [21], were ‘α’ was a factor that determined the height of the threshold. Each time an averaged value was above the defined threshold the data was marked as an outlier. The factor and the width of the moving average window were varied in order to retrospectively determine the most optimal set of parameters that lead to the best algorithm for outlier detection: ‘α’ was varied between the constants 0.5 and 3.0 in steps of 0.1 and ‘δ’ was varied between 0.5 and 3 s in steps of 0.01 s. All possible combinations between both parameters were used.

The time points that were identified as actual events of therapeutic balance assistance (captured by both the force sensors as well as other locations on the body) were loaded into the acceleration dataset and compared with the outliers that were detected by the algorithm. Each outlier was classified as a True Positive (TP; both therapist and algorithm classify point in time as an event of balance assistance), False Negative (FN; therapist provides assistance, but algorithm does not find outlier) or False Positive (FP; therapist does not provide assistance, but algorithm finds outlier). Additionally, the Positive Predictive Value (PPV = TP/(TP + FP)*100) and True Positive Rate (TPR = TP/(TP + FN)*100 were calculated for each patient individually and on a group level, indicating the ability of the algorithm to identify the events of balance assistance correctly.

Optimal settings were defined as the best combination of ‘α’ and ‘δ’, leading to the highest score of PPV and TPR at the same time. Optimal settings were determined by using the data of four, randomly selected subjects. The data of the remaining four subjects were used to validate these settings, and to test whether these settings still resulted in high PPV and TPR values for other subjects.

## Results

Eight measurement sets were performed with the included patients. None of the subjects fell during the measurements. A total of seven different therapists provided physical balance assistance during the training sessions. Patient characteristics as well as scores on clinical measures are presented in Table 1.

**Table 1:** Patient characteristics and measured walking speed during the trials

Patient ID (#)	Gender (M/F)	Age (yrs)	Weight (kg)	Therapist ID (#)	BBS (pnts)	FAC (pnts)	10MWT (m/s)	MI (MI leg) (pnts)		DGI (pnts)	Walking speed during trials (m/s)
1	M	60	81	1	41	3	0.17	51	(42)	12	0.22
2	M	56	91	1	38	3	0.46	42	(28)	10	0.38
3	M	64	76	2	32	3	0.31	81	(42)	14	0.31
4	M	54	80	3	46	4	0.85	107	(53)	–	0.54
5	M	58	82	4	47	4	0.47	62	(34)	13	0.33
6	M	68	85	5	34	3	0.27	77	(48)	–	0.28
7	M	58	79	6	44	2	0.4	132	(57)	16	0.20
8	M	49	98	7	46	4	0.4	153	(83)	12	0.30
Median (IQR)	–	58 (5.5)	82 (7)	–	43 (9)	3 (1)	0.40 (0.16)	79 (54)	45 (14)	13 (1.8)	0.31 (0.07)

Scores on clinical tests and the self-selected chosen walking speed are presented for each patient individually

### Balance-assisting force characteristics based on the force sensor data

Characteristics of the provided therapeutic correction forces are presented in Table 2. Median number of balance-assisting events during the total measurement set of each patient was 3 times (0.6) (median (IQR)). Median distance walked during this time span was 35 m (21) (11.5 m (18.3) walked per event of balance assistance, median (IQR)).

**Table 2:** Characteristics of therapeutic balance assistance during measurement trials

Patient ID (#)	Total events of balance assistance (#)	Travelled distance (m)	Travelled distance / event (m)	Mean peak force /event (ML-axis) (N) (% body weight)		Mean peak force / event (SI-axis) (N) (% body weight)		Mean peak force / event (AP-axis) (N) (% body weight)		Mean Duration (s)	Mean Impulse (Ns)	Location of event / number of sensors hit (#)		
												Both sensors	One Sensor	Other
1	6	28	5	12.3	(1.5)	2.2	(0.27)	1.2	(0.15)	0.72	2.5	4	1	1
2	3	42	14	13.6	(1.5)	1.9	(0.21)	1.3	(0.14)	0.99	6.3	1	1	1
3	3	28	9	28.9	(3.8)	5.1	(0.67)	2.2	(0.29)	0.95	13.9	0	1	2
4	1	21	21	–	(−)	–	(−)	–	(−)	–	–	0	0	1
5	1	63	63	–	(−)	–	(−)	–	(−)	–	–	0	0	1
6	7	42	6	18.1	(2.1)	1.4	(0.16)	2.2	(0.26)	1.7	12.5	7	0	0
7	3	15	5	6.5	(0.82)	1.8	(0.23)	4	(0.51)	1.2	4.2	1	2	0
8	3	100	33	25.5	(2.6)	1.1	(0.11)	2.4	(0.24)	1.9	13.1	0	3	0
Median (IQR)	3 (1.3)	35 (21)	11.5 (18.3)	15.9 (11)	(1.8) (0.98)	1.9 (0.7)	(0.22) (0.08)	2.2 (0.8)	(0.25) (0.11)	1.1 (0.6)	9.4 (8.2)	–	–	–

ML = medio-lateral, SI = superior-interior, AP = anterior-posterior. Missing data is indicated by an ‘-‘: therapists did not provide assistance at the location of the force sensors in these cases. Characteristics of these events could therefore not be calculated

Corrective forces were mainly provided over the medio-lateral (ML) axis (frontal plane): Roughly 80% of the total force that was used is provided in this plane, representing 2% of the average body weight of the subjects. Around 10% (±0.2% of the patient’s body weight) of the total force was provided over both the anterior-posterior (AP) and superior-inferior (SI) axes. Seventy-eight percent of the total amount of events of balance assistance was located at the side of the pelvis (either on one or on both force sensors on the iliac crest). Other locations that were used, were the trunk and shoulder of the patient. The median duration of therapeutic assistance was typically 1.1 s (0.6) and the median impulse per event over the ML-axis was typically 9.4Ns (8.2).

**Fig. 4:**
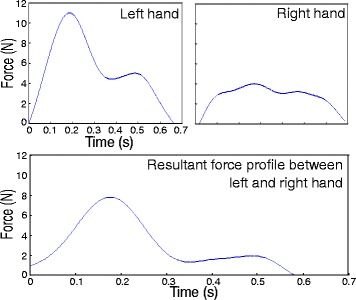
Typical example of a force profile over time. Two hands were used during this event. Force profiles of both the left and right hand have been presented in the figure, as well as the resultant force between both hands. A positive value on the y-axis indicates a pushing force by the therapist. In this particular example, the subject is pushed towards the right

Typical examples of therapeutic balance-assisting force profiles are shown in Fig. 4. Here, two hands have been used for the application of corrective force. The left hand exerts the largest amount of force on the body. The right hand of the therapist exerts a smaller amount of force on the body. It is used to provide stability to the pelvis of the subject and it helps the therapist in controlling the application and release of the corrective forces.

### Predicting the timing of therapeutic balance assistance based on acceleration data of the sacrum

Based on four randomly selected subjects (patient ID = 1, 4, 5 and 7), there were several combinations of window width ‘δ’ and factor ‘α’ that lead to similar PPV and TPR score. In all cases, ‘δ’ was 2.5 s and ‘α’ could be chosen as a constant value between 0.8 and 1.3, all leading to the same PPV and TPR values. With these algorithm settings, a PPV of 73% and a TPR of 80% was obtained on a group level. Individual PPV and TPR scores ranged between 67% and 100%.

The algorithm was validated by applying these settings (with ‘α’ chosen as a constant of 1.0) to the remaining four subjects (patient ID = 2, 3, 6, 8). PPV and TPR values increased in this case to 87% and 81% respectively, on a group level. PPV and TPR scores of the individual subjects remained between 67% and 100%. Both the scores on an individual level as well as on a group level are presented in Table 3 and Table 4, for both groups.

**Table 3:** Individual and group scores of subjects that were randomly selected to be in the development group of the algorithm

Patient ID (#)	TP (#)	FN (#)	FP (#)	PPV (%)	TPR (%)
1	4	2	1	80	67
4	1	0	0	100	100
5	1	0	0	100	100
7	2	1	1	67	67
Group total	8	3	2	73	80

Scores are presented as the summed total of all measurements trials within a patient

**Table 4:** Individual and group scores of the subjects that were part of the validation group of the algorithm

Patient ID (#)	TP (#)	FN (#)	FP (#)	PPV (%)	TPR (%)
2	2	1	1	67	67
3	3	0	0	100	100
6	5	2	0	100	71
8	3	0	1	75	100
Group total	13	3	2	87	81

Scores are presented as the summed total of all measurements trials within a patient

**Fig. 5:**
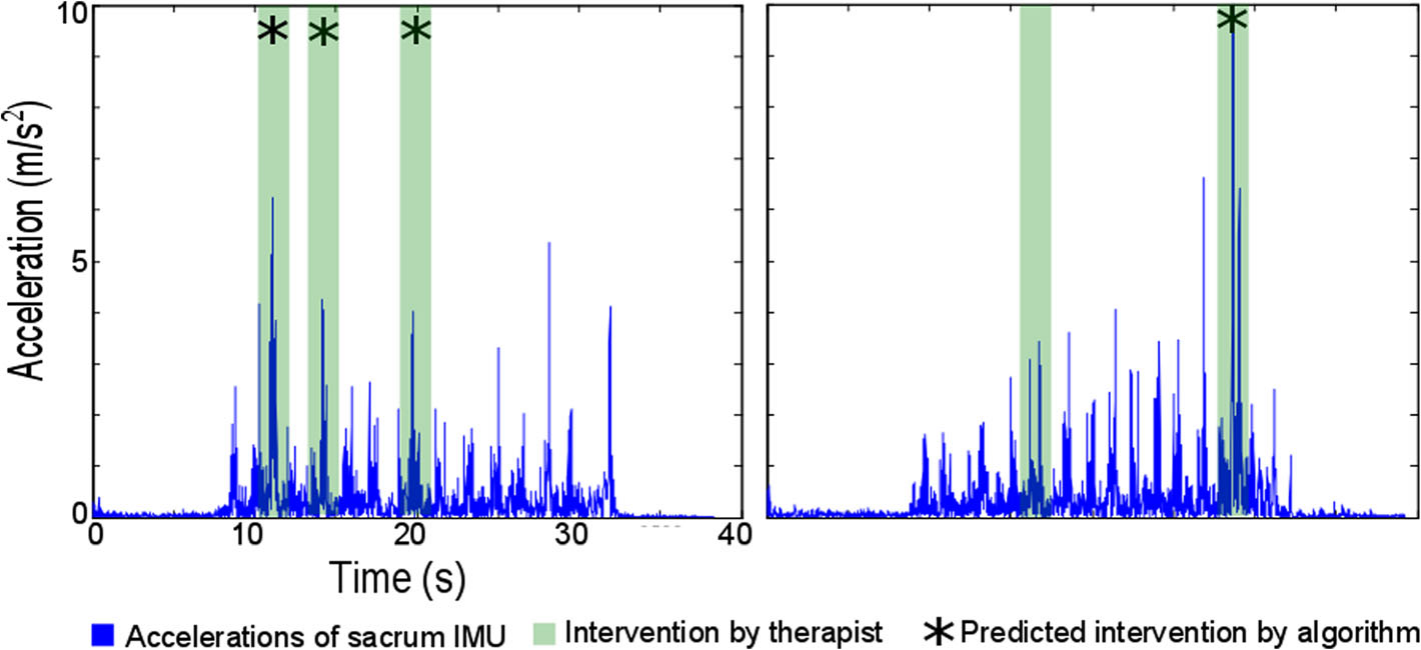
Typical examples of acceleration signals over time in relation to the actual and predicted events of balance assistance by the therapist and the algorithm. Typical examples of acceleration signals over time (blue). With the events of therapeutic assistance marked in green and the outliers detected by the algorithm marked with an asterisk. Note that the algorithm calculates average values of the data (by using the moving average filter) and compares them with the specified threshold. Therefore, several high peaks of the original sacral signal that are shown in blue are not detected by the algorithm as events of balance assistance, as they do not last long enough to result in averaged values that are high enough to exceed the threshold. Characteristics of the corrective forces are as follows: Left graph: Right graph: Event #1 (TP) / #2 (TP) / #3 (TP) Event#1 (FN) / #2 (TP) Peak Force 8.1 N / 6.5 N / 5.1 NPeak Force: unknown / 48 N Location: Iliac Crest / Iliac Crest / Iliac Crest Location: Shoulder / Iliac Crest Duration: 0.42 s / 1.3 s / 1.2 s Duration: unknown / 0.57 s

Two typical examples of an acceleration signal over time are shown in which all balance-assisting events were correctly classified (Fig. 5, left) or in which one of two events was not detected (Fig. 5, right). Note that the events that were not detected, the therapeutic assistance force was applied at the shoulder.

## Discussion

This paper is the first to present insights into the behavior of therapists regarding manual physical balance assistance to stroke survivors during gait training in terms of force characteristics and prediction of the timing. We aimed to provide a quantitative dataset on the balance-assisting force characteristics and provide insight in the timing of these events, as such information is of importance in the technical development of (robotic) devices that support balance. As therapist integrate knowledge on many aspects of the patient, the results of this paper should be interpreted as a first step towards the analysis of this complex behavior. Regarding the timing of the balance-assisting events, we developed an algorithm that is able to predict therapeutic events, solely based on the acceleration signal of the patient’s sacrum during a gait training session. Prediction scores remain high, even when different therapists and patients were analyzed with the same algorithm settings. Additionally, the results that were shown in the quantitative dataset imply that the therapists in this study in general use similar balance-assisting strategies while physically supporting the patients. For instance, correction forces were relatively low in all cases, corrective forces were mainly provided over the ML axis (80%) and the spread in duration was rather small. The quantitative results in this paper might best be interpreted as guidelines, rather than being fixed numbers, especially as this dataset is based on only eight subjects.

The complex clinical decision-making process of therapist in determining whether or not to apply a balance-assisting force, might explain PPV and TPR scores that are lower than 100%, indicating that the algorithm was not able to identify all therapeutic events correctly. Therapists integrate information such as the patient’s muscle strength, reaction times, fatigue state and fall history in order to determine whether or not to provide balance assistance. Although the developed algorithm in our study already shows high prediction scores, a more elaborated or altered measurement set-up and algorithm might be needed to better approach the complex clinical decision-making process of therapists. Studies that focus on the prediction of falls, such as in the study by Li et al. [22] or Sim et al. [23] report PPV rates of 80% to 90%, and show that by adding more accelerometers or additional gyroscope sensors to the body (e.g. head or waist) the performance of the algorithm could be further increased. It was shown by, among others, Li et al. [22] and Bourke et al. [24] that performance of fall detection systems can be even further increased when adding gyroscope data to the algorithm, supplementary to acceleration data. This might specifically be beneficial when a large variety of ADL tasks need to be classified during gait training [25, 26] and an adequate distinction between the body movements is needed.

Although therapists aim to treat patients with the best possible care, there could have been situations in which the therapist was too careful and therapeutic balance assistance was provided at times when this was not actually needed. These situations could have led to FN scores at the side of the algorithm as no deviations would then be visible in the measured signal. This example indicates the complexity in interpreting FN and FPscores. For (robotic) devices, both FN as well as FP values should be as low as possible: missing a critical balance-assisting event at the side of the patient could lead to an actual fall when no safety harness is present, whereas the system might not measure up to the principles in error-based training when assistance is provided too soon, thereby letting the patient not experience the boundaries of his abilities. By changing the algorithm settings (‘α’ and ‘δ’) that are presented in this study, one can adapt the error-based training component: allow more or less movement freedom, depending on the abilities of the patient. It should be emphasized that the present research and the developed algorithm serves as a first step towards this process. No (robotic) devices that focus on balance assistance are known that use a control mechanism that tries to mimic the complex behavior of therapist, whereas this might be of great importance in the acceptance and effectiveness of such a device. As a first step in this development, the developed algorithm and the used measurement set-up were kept as simple and intuitive as possible.

Even though the algorithm proves to have a good ability to predict therapeutic balance-assisting events correctly, it does not directly validate that sacral acceleration is wholly responsible for the therapist balance-assisting responses. In fact, a different contact location was reported in 22% of the corrections, even though therapists were specifically instructed to aim for the force sensors on the iliac crest. Beside the argument that this could be just common practice of therapists, the typical example of Fig. 5 demonstrates that a different trigger (arm sway of the patient in order to restore balance) other than sacral accelerations, causes the therapist to apply corrective forces to the body. Although with the use of the present measurement setup it was attempted to capture a conventional gait training session as realistically as possible, restrictions in the used measurement set-up have limited the recording of the events that were not located on the force sensors. Consequently, these events were not taken into account in calculating median values. Yet, the used measurement set-up did not affect the number of balance-assisting events in any way, nor did it affect the timing of the events. Even though the use of force sensors logically deviates from a conventional clinical setting, none of the therapists indicated that they were forced to balance the patient at a location at which it was inconvenient for them. It was therefore expected that regarding the specific location of providing balance assistance, the measurement set-up did not significantly affect the behavior of the therapists. Logically, other methods could have been chosen to capture the corrective forces by therapists. For instance the use of force gloves that would allow the capturing of all therapeutic forces, regardless of the contact location on the body. However, most known force gloves [27, 28] are still in the development stage and/or have a limited amount of sensors on the glove, thereby introducing the risk that not all contact force between therapist and patient would be measured by the glove. Additionally, using these gloves would introduce the risk of having only one measurement from a specific body location, thereby restricting the ability to generalize the results. As the main goal of this manuscript was to provide first insights into the behavior of therapists, we have chosen to focus specifically on one location on the body at this stage of the research. Additionally, thickness of the force sensor prevents any contact between the therapist’s hand and the patient’s body: thereby ensuring that all force is captured by the force sensor.

Furthermore, in 38% of all balance-assisting events located at the iliac crest, the hands of the therapist hit only one sensor, even though two hands were used for the balance-assisting event in these cases. This affected the calculation of the resultant force, as this was calculated as the difference between both hands. It caused an undershoot of the resultant force in some cases and overshoot in others. Yet, it is believed that the presented median corrective force is still a good representation of the average force profile that is used during a gait training session.

### Limitations

Although this study was conducted with care, a couple of limitations can be identified. First of all, it should be noted that a certain inaccuracy exists in the time synchronization between both measurement systems. Both measurement systems were synchronized by pressing a switch (triggering the IMU software to start recording) against one of the force sensors (leading to a peak value in this data signal). Force data was cut afterwards in MATLAB, based on this synchronization peak. Even though it was intended by the researchers to shorten the duration of the peak value as much as possible, a small inaccuracy remained in the time synchronization between both systems. Secondly, a delay might exist between the instability of the subject and the timing of the balance-assisting event of the therapist, i.e. between a peak in the recorded acceleration data and a peak in the recorded force data. Both peaks do not occur exactly at the same time, mainly due to the reaction time of the therapist. Yet, it was assumed that the total time inaccuracy between both systems was no more than ±0.5 s: When the actual balance-assisting event and the detected outlier were a maximum of ±0.5 s apart, the outlier was classified as a TP.

Furthermore, the algorithm is based on data of four patients and four therapists, and is validated with data of the other four patients and three therapists. Positive Predictive Values and True Positive Rates remain high in the validation process, even though measurements of different subjects and patients were then included. This positively suggests that the algorithm is valid for a random group of therapists and/or patients, rather than being restricted to the measured group of subjects. However, it must be kept in mind that the optimal parameter settings (‘α’ and ‘δ’) that determined PPV and TPR scores, were in both cases obtained by using a group of (four) subjects with rather homogeneous patient characteristics. These setting might not be the most optimal settings when focusing on individual subjects, or even a single measurement trial. This is confirmed by the finding that for individual patients, PPV and TPR scores vary between 67% and 100%. Therefore, the present research only functions as a first step in the process of capturing the complex behavior of therapists.

### Future work

The algorithm that was developed and the results of the balance-assisting force characteristics provide a good first impression of the requirements that can be set to (robotic) devices that support balance. Future work should first of all increase the number of subjects to further specify these requirements. A larger and more heterogeneous group of FAC 3 patients should be included for these measurements and the patients should perform additional tasks during the measurements: step over objects, 180 degree turns, variable step length, etcetera.

Furthermore, increasing the number of subjects, therapists and measurements would allow to specify inter-therapist variability. Although the quantitative results in this study imply that therapists in general use similar balance-assisting strategies, specific details on this matter might be of interest while developing robotic training devices, for instance in relation to specific walking tasks [29]. Although it must be kept in mind that force characteristics might differ for each individual subject, training session and even for each intervention, increasing the number of subjects might enable the classification of balance-assisting events, thereby provide more insight into the aspect of inter-therapeutic variability.

In order to capture additional aspects of the complex behavior of therapists, it might be of interest to focus on verbal and sensory cues of therapists, to obtain characteristics of other body locations (such as shoulder or trunk), and to specify the directions of the balance assistance in relation to the gait cycle. For instance, it has now been observed from video analysis, in correspondence with the recorded data, that patients mainly fall sideways or to the back, and the therapist consequently pushes the patient upwards, to the front and over the medio-lateral axis. It might be of interest to study the ML balance assistance in relation to the gait cycle at the moment of intervening, and in relation to the affected side of the subject.

## Conclusion

The goal of this study was to present a quantitative dataset on therapeutic balance assistance and predict the timing of these events. A threshold-based algorithm was developed that was able to predict timing of therapeutic balance assistance events with a Positive Predictive Value of 87% and a True Positive Rate of 81%.

Quantitative data showed that patients on average received a balance-assisting corrective force every 11.5 m and that instability mainly occurred in the frontal plane. They received force corrections by the therapists of around 2% of their total body weight, with a median duration of 1.1 s and a median impulse of 9.4 Ns. This study represents the first step in the analysis of complex behavior of physical therapists during gait training and this information can serve as input for the development of (robotic) devices that support balance.

## Abbreviations

10MWT: 10 meter walking test
AP: Anterior-Posterior
BBS: Berg balance scale
COM: Center of mass
DGI: Dynamic Gait Index
FAC: Functional ambulation category
FN: False negative
FP: False positive
IMU: Inertial measurement unit
IQR: Interquartile range
MI: Motricity index
ML: Medio-lateral
PPV: Positive predictive value
Q1-Q4: Quartile 1 – Quartile 4
SI: Superior-inferior
TP: True positive
TPR: True positive rate
